# Medical Teaching and Opportunities in Philadelphia

**Published:** 1857-11

**Authors:** 


MEDICAL TEACHING AND OPPORTUNITIES IN PHILADELPHIA.—It is improbable that many of those not directly interested in medical instruction know the extent of its ramifications in this city at the present time. Besides the four graduating Schools, an immense amount of talent, experience, and industry are engaged in supplying what, though auxiliary, can hardly be called subordinate portions of medical instruction, since they are, in some instances, parallel in importance to those of the Colleges. We propose to name some of the principal extra-collegiate facilities afforded here to the student or recent graduate.

First, we have the Pennsylvania Hospital, in which clinical instruction is given twice every week through the whole year, and daily during a portion of the summer. The present staff of this admirable institution consists of Drs. G. B. Wood, W. Pepper, W. W. Gerhard, and J. J. Levick, attending Physicians, and Drs. G. W. Norris, E. Peace, J. Neill, and J. Pancoast, attending Surgeons; besides a well-selected corps of resident surgeons and physicians. The advantages of this hospital are especially great for the study of practical surgery; the number of accidents alone brought into its wards, averaging, now, two per diem. The fine Medical Library of the Pennsylvania Hospital, the largest in the country, is accessible to students as well as to practitioners.

Wills' Hospital for Diseases of the Eye and Limbs, contains a large number of ophthalmic patients; while its *ambulatorium* exhibits, also, the treatment of a host of out-door applicants. No clinique is advertised at this hospital; but private students are admitted on prescribing days, to see cases and operations; and Drs. A. Hewson and W. Hunt conduct, during their terms, a combined course of demonstrative instruction. The other surgeons of Wills' Hospital, Drs. S. Littell and E. Hartshorne, receive pupils, but do not lecture.

The Obstetric Institute, established many years ago by Dr. Joseph Warrington, in connection with the Lying-in Charity of the Philadelphia Dispensary, is now conducted by Drs. E. Wilson and J. M. Corse. A very large number of cases of labor is attended annually under their supervision, by gentlemen connected with their classes; who also receive the benefit of practical lectures and exercises, and of a weekly clinique for diseases of females. Four courses of instruction are given every year in this institute.

Of a somewhat similar character are the private schools of Practical Obstetrics conducted, separately, by Dr. R. A. F. Penrose and Dr. E. McClellan.

The Western Clinical Infirmary is intended to realize the advantages of the classification of diseases and injuries for special treatment and study. Its medical officers are Drs. J. Klapp, O. H. Partridge, L. Turnbull, C. P. Turner, J. Aitken Meigs, G. R. Morehouse, D. D. Clark, R. L. Madison, W. H. Gobrecht, and E. McClellan. A series of lectures is contemplated by these gentlemen, in connection with the cliniques of the Infirmary.

Several of the more private courses of Lectures upon practical subjects are also illustrated by the examination and treatment of cases. Such are those of Dr. W. W. Gerhard on Diseases of the Lungs and Heart; Dr. J. Da Costa on Physical Diagnosis; Dr. J. H. Brinton on Operative Surgery; Dr. R. L. Madison on Surgery; Dr. C. S. Bishop on Practical Surgery; Dr. J. H. Packard on Minor Surgery and Bandaging;

Dr. J. Darrach on Diseases of the Urinary Organs, and Dr. L. Turnbull on Affections of the Eye and Ear.

Two of the gentlemen already mentioned are connected with the Association for Medical Instruction, whose lectures are confined to the summer season. The faculty of this, the oldest organization for extra-collegiate medical teaching in this city, now consists of Dr. Bridges, Lecturer on Chemistry; Dr. West, on Materia Medica; Dr. Wallace, on Anatomy; Dr. Keating, on Obstetrics; Dr. S. W. Mitchell, on Institutes of Medicine; Dr. Da Costa, on Practice; and Dr. Hewson, on Surgery.

Of private schools of Anatomy there are at least two: those of Dr. Agnew and of Dr. Forbes; besides a course on Pathological Anatomy, by Dr. Woodward. A course on the Microscope has been, also, given by Dr. J. C. Morris.

Practical Chemistry is systematically taught, with every facility, in several laboratories, of which we may name that of Mr. Booth as the most widely known.

Botany, with the aid of excursions into the country during the summer, is taught by Harlan Coultas and by Prof. Ennis.

By no means among the least important among the aids to the student or graduate, is the School of Practical Pharmacy, conducted by E. Parrish; from an introductory lecture delivered in which we have taken much of the matter of the above sketch. The courses in the Philadelphia College of Pharmacy are accessible to medical students as well as to druggists. Its faculty consists of Prof. R. Bridges, who lectures on Chemistry; Prof. R. P. Thomas on Materia Medica, and Prof. W. Procter on Pharmacy.

We ought not to omit allusion, in this brief summary, to the associations for *examining* students upon the different departments of medical study, of which several exist in connection with the different schools: nor to the combinations for affording office and other facilities for prolonged instruction, in which a number of prominent medical gentlemen are engaged. Nor, finally, is it a trifling advantage to the zealous student to have access to the magnificent collection of the Academy of Natural Sciences, to which his matriculating ticket will admit him two days in the week.

